# Affective Determinants of Physical Activity: A Conceptual Framework and Narrative Review

**DOI:** 10.3389/fpsyg.2020.568331

**Published:** 2020-12-01

**Authors:** Courtney J. Stevens, Austin S. Baldwin, Angela D. Bryan, Mark Conner, Ryan E. Rhodes, David M. Williams

**Affiliations:** ^1^Department of Psychiatry, Dartmouth-Hitchcock Medical Center, Geisel School of Medicine, Dartmouth College, Lebanon, NH, United States; ^2^Department of Psychology, Southern Methodist University, Dallas, TX, United States; ^3^Department of Psychology and Neuroscience, University of Colorado Boulder, Boulder, CO, United States; ^4^School of Psychology, University of Leeds, Leeds, United Kingdom; ^5^School of Exercise Science, Physical & Health Education, University of Victoria, Victoria, BC, Canada; ^6^Department of Behavioral and Social Sciences, Department of Psychiatry and Human Behavior, School of Public Health, Brown University, Providence, RI, United States

**Keywords:** affect, physical activity, exercise, Affect and Health Behavior Framework, affective response, incidental affect, affect processing, affectively charged motivational states

## Abstract

The literature on affective determinants of physical activity (PA) is growing rapidly. The present paper aims to provide greater clarity regarding the definition and distinctions among the various affect-related constructs that have been examined in relation to PA. Affective constructs are organized according to the Affect and Health Behavior Framework (AHBF), including: (1) *affective response* (e.g., how one feels in response to PA behavior) to PA; (2) *incidental affect* (e.g., how one feels throughout the day, unrelated to the target behavior); (3) *affect processing* (e.g., affective associations, implicit attitudes, remembered affect, anticipated affective response, and affective judgments); and (4) *affectively charged motivational states* (e.g., intrinsic motivation, fear, and hedonic motivation). After defining each category of affective construct, we provide examples of relevant research showing how each construct may relate to PA behavior. We conclude each section with a discussion of future directions for research.

## Introduction

The relationship between regular physical activity (PA) and health is indisputable (Rhodes et al., 2017; Warburton and Bredin, 2017; Guthold et al., 2018; Mandsager et al., 2018; Piercy et al., 2018). Data collected from population-based prospective cohort studies suggest that engaging in 1–5 times the recommended minimum amount of weekly PA is associated with a 31–39% reduced risk for all-cause mortality (Arem et al., 2015). Furthermore, the economic consequences of physical inactivity are exorbitant. A recent “global analysis” conservatively estimated the annual cost of physical inactivity to be $53.8 billion worldwide (Ding et al., 2016).

Both national and international PA guidelines specify that adults should “move more and sit less” and that “some PA is better than none” (World Health Organization, 2010; Piercy et al., 2018). However, to achieve substantial health benefits, adults aged 18–64 should do at least 150 min of moderate-intensity aerobic PA or 75 min of vigorous-intensity aerobic PA throughout the week (or an equivalent combination of both). Bouts of aerobic PA should be performed in sessions of at least 10 min duration and muscle strengthening activities should additionally be done 2 or more days per week and involve all major muscle groups (World Health Organization, 2010; Piercy et al., 2018). Despite wide dissemination of these guidelines, only 23% of adults achieve guidelines for both aerobic and muscle strengthening PA, and 44% do not meet either guideline (USDHHS, 2018).

There is considerable public health incentive to identify strategies for increasing PA participation and maintenance in the population. A current topic of debate in the literature concerns the extent to which social cognitive theories, which have a long history of application in PA intervention research, have “outlived their usefulness” (Sniehotta et al., 2014; Linde et al., 2016), and the extent to which affective/experiential factors, historically omitted from dominant theoretical models, might help to elucidate the “intention-behavior gap” observed in PA research (Rhodes and de Bruijn, 2013; Sheeran and Webb, 2016; Rhodes and Gray, 2018). As such, efforts to understand who will, and who will not maintain PA over time have increasingly focused on affective correlates and determinants of behavior (Williams and Evans, 2014; Conner et al., 2015; Rhodes and Kates, 2015; Conroy and Berry, 2017; Ekkekakis, 2017; Brand and Ekkekakis, 2018; Williams et al., 2018; Ekkekakis and Brand, 2019).

**Figure 1:**
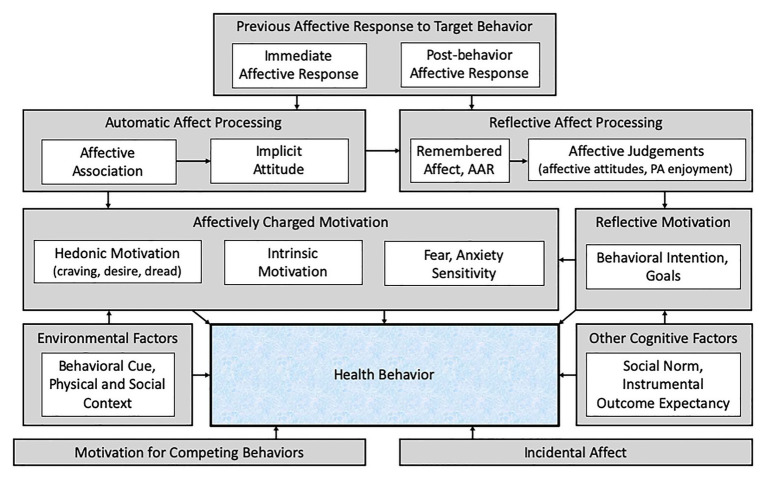
The Affect and Health Behavior Framework (adapted from Williams and Evans, 2014). AAR, anticipated affective response; PA, physical activity. Prior affective responses to a health behavior (physical activity) influence both automatic and reflective affect processing, which informs both affectively charged and reflective motivation to perform (or avoid) the behavior. Affect unrelated to the target behavior experienced throughout the day (incidental affect) also influences behavior, as do other cognitive factors, environmental factors, and motivation to perform other competing behaviors.

According to the Affect and Health Behavior Framework (AHBF), first proposed by Williams and Evans (2014), affective correlates and determinants of health behavior can be divided into four categories with respect to their association with the target behavior (see Figure 1). These are (1) affective response, which is how one feels while performing a behavior and/or immediately after completing a target behavior; (2) incidental affect, which is how one feels throughout the day, outside the context of the target behavior; (3) affect processing, which concerns cognitive processing of previous affective responses and may be automatic (affective associations and implicit attitudes) or reflective (anticipated affective response, remembered affect, and affective judgments); and (4) affectively charged motivation, which includes motivational states that have their basis in past affective responses to PA and motivational states that include and/or have a basis in past affective responses to PA and are elicited through both automatic (hedonic motivation) and reflective (intrinsic motivation and fear) processing pathways. Numerous studies have explored the roles that these various affective categories play in predicting and/or promoting PA; however, at present, there have been no attempts to summarize these approaches within a single review.

The present article aims to apply the AHBF to the domain of PA and provide a selective review of relevant studies on affect-related correlates and determinants of PA. It is not the intention of this article to present a systematic and quantitative review of research on all affect-related factors but rather to distinguish among various affect-related constructs and provide an illustration of how each construct may relate to PA behavior. Consistent with the AHBF, the focus of the present review is on affective factors as putative determinants of PA behavior. That is, we are interested in PA behavior – not affect – as the dependent variable.

### Key Terminology

Before proceeding, a note on terminology is warranted. We use “PA” as a superordinate term to summarize and integrate findings across studies in which either purposeful exercise or PA (encompassing all daily kinesthetic movement including, but not limited to, activities performed for the purpose of exercise) was examined as the outcome of interest. Further, we use the superordinate term “affect-related” to refer to the full range of constructs represented by all four AHBF categories (see Table 1). We do this to increase ease of readability as a wide range of affective states have been measured with respect to PA and relatedly use of affective terminology has varied across the literature (Ekkekakis et al., 2011; Ekkekakis, 2013). Conversely, the term “affect” will only be used to refer to affect *per se*, which includes core affect, emotions, and moods. The first two categories of constructs in the AHBF – affective response to PA and incidental affect – constitute affect *per se*, whereas the second two categories represent affect-related cognition and affectively charged motivation, respectively.

**Table 1:** Affective determinants of physical activity (PA) represented in the Affect and Health Behavior Framework (AHBF).

**Affective response**	
Definition	How one feels while performing and/or immediately after completing a target behavior
Key features	Affect, *per se*, can only be experienced *in vivo*
Constructs measured in relation to PA	Core affect (valence and arousal)
Assessment	Assessment by self-report (e.g., core affective valence): *Choose the number that best describes how you feel right now… “very bad”* (−5) – *“very good”* (+5) Measures may be administered in-person or remotely (*via* ecological momentary assessment)
**Incidental affect**	
Definition	How one feels throughout the day, unrelated (incidental) to the target behavior
Key features	Affect, *per se*, can only be experienced *in vivo*
Constructs measured in relation to PA	Core affect, specific affectively charged states (fatigue), moods, and emotions
Assessment	Assessment by self-report (e.g., negatively valenced affect): *How stressed are you feeling right now… “not at all stressed”* (+1) – *“extremely stressed”* (+5) Data often collected using ecological momentary assessment
**Affect processing**	
Definition	Cognitive processing of previous affective responses
Key features	Occurs through both automatic and reflective pathways
Constructs measured in relation to PA	Automatic affect processing constructs: affective associations and implicit attitudes Reflective affect processing constructs: remembered affect, anticipated affective response, and affective judgments
Assessment	Automatic affect processing assessment by reaction-time tasks (e.g., implicit attitudes): Participants respond to PA-related word or image cues paired with affective descriptors Automatic affect processing assessment by self-report (e.g., affective associations): *When I consider physical activity, I feel happy… “strongly disagree”* (+1) – *“strongly agree”* (+5) Reflective affect processing assessment by self-report (e.g., affective attitudes): *For me, engaging in moderate-vigorous PA would be…. “Very unpleasant”* (0) – *“Very pleasant”* (+100)
**Affectively charged motivation**	
Definition	A motivational state that includes and/or has its basis in past affective responses to PA
Key features	Occurs through both automatic and reflective pathways The counterpart to reflective motivation (i.e., goals and intentions)
Constructs measured in relation to PA	Hedonic motivation (automatic desire/wanting vs. dread); fear/anxiety sensitivity
Assessment	Intrinsic motivation is the mostly commonly assessed construct in this category and is assessed by self-report: *I get pleasure and satisfaction from participating in exercise … “strongly disagree”* (+1) – *“strongly agree”* (+5) Hedonic motivation (automatic desire/wanting vs. dread) has been assessed with one-item self-report measures but can also be assessed *via* neurobiological assays (e.g., “wanting” as dopamine release). More research is needed to distinguish self-report of hedonic motivation from reflective wanting or dread, which may be based on deliberate consideration of the long-term consequences of exercise (e.g., health benefits) and, thus, is not consistent with hedonic motivation.

PA, physical activity. Examples listed under assessments are intended to provide a brief illustration of how affect-related constructs from each category of the AHBF are commonly assessed in research. Examples are by not exhaustive.

**Figure 2:**
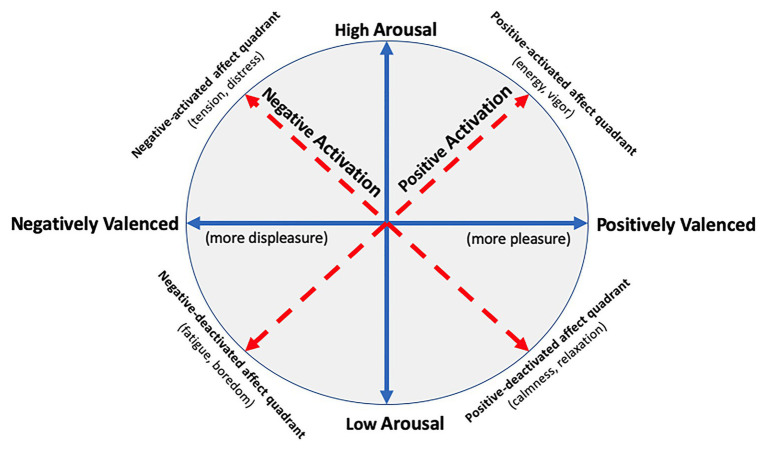
Core affect as depicted by the unrotated and rotated circumplex models (adapted from Ekkekakis, 2013). Solid arrow lines (blue) represent the dimensions of the unrotated circumplex model of affect (Russell, 1980), and dotted arrow lines (red) represent the dimensions of the rotated circumplex model of affect (Watson and Tellegen, 1985) using the revised labels for each bipolar dimension (Watson et al., 1999). As depicted, the dimensions of the unrotated circumplex model generate four quadrants and certain measures of affect, such as the Physical Activity Affect Scale (PAAS; Lox et al., 2000), measure constructs that map onto these quadrants.

We also want to clarify terminology regarding core affect, as core affect is commonly used when measuring affect *per se* in relation to PA, and at times, there has been confusion about how to describe and label core affect. To summarize briefly, Russell (1980) proposed a circumplex model to describe core affect whereby two neurophysiological systems, valence (pleasure-displeasure) and arousal (energy-lethargy), give rise to all other feeling-based experiences (see Figure 2). Later, Watson and Tellegen (1985) proposed a rotated circumplex model of affect, which rotates the two core affective dimensions 45°, yielding two orthogonal dimensions: a positive activation dimension ranging from “positive activated affect” (e.g., energy and vigor) to “negative deactivated affect” (e.g., fatigue and boredom) and a negative activation dimension ranging from “negative activated affect” (e.g., tension and distress) to “positive deactivated affect” (e.g., calmness and relaxation).

Initially, Watson and Tellegen labeled the dimensions of the rotated circumplex model [and the associated Positive and Negative Affect Schedule (PANAS); Watson et al., 1988] “positive affect” and “negative affect” because they believed the constructs were best defined by their high-arousal poles. There was significant debate in the literature regarding this choice (Feldman Barrett and Russell, 1998) and later Watson et al. (1999) published a paper that renamed the two dimensions as “positive activation” and “negative activation.” Despite this, many authors continue to use the original labels for the rotated circumplex model’s dimensions (i.e., “positive affect” and “negative affect”). This has led to the mistaken belief that the positive activation and negative activation dimensions of the rotated circumplex model are meant to reflect pure pleasure and displeasure, respectively, and that pleasure and displeasure may be conceptually orthogonal (see Ekkekakis, 2013). Therefore, although potentially more cumbersome, when referring to the valence dimension of Russell’s (1980) unrotated circumplex model, we use the terms “positively (or negatively) valenced affective states” or “positive (or negative) shifts in affective valence” to distinguish these affective concepts from the (original) “positive affect” and “negative affect” dimensions of the Watson and Tellegen’s rotated circumplex model.

## Affective Response to PA

Affective response to PA is how one feels in response to PA behavior. By this definition, affective response can only be experienced *in vivo* and, therefore, it should be measured while a participant completes his or her assigned PA on a treadmill (or other type of equipment). For example, a researcher might ask a participant to provide a rating on an affective response self-report measure, such as the Feeling Scale (Hardy and Rejeski, 1989), every few minutes during and immediately following the laboratory-based PA task (i.e., “Please choose the number that best describes how you feel right now on a scale from −5 = *very bad* to +5 = *very good*”). More recently, increasingly sophisticated and commercially available technology has made it possible for researchers to query participants’ affective responses to PA in their natural living environments, in-real-time, through the use of ecological momentary assessment (EMA).

When the focus is on affective response to PA as a determinant of future PA, rather than as an outcome in its own right (e.g., PA as a treatment for depression or anxiety), it is, consistent with psychological hedonism, core affective valence (pleasure vs. displeasure; Russell, 1980) that is the most important aspect of affective response. Alternatively, there are no obvious theoretical or conceptual indications as to what specific affective states – sadness or embarrassment, joy or tranquility – might, in the context of affective response to PA, add to the prediction of future PA behavior beyond positive vs. negative affective valence (Ekkekakis and Petruzzello, 2000). Thus, much of the literature on affective response to PA as a putative determinant of future PA has, appropriately, emphasized core affective valence.

In any case, for purposes of understanding the potential influence of affective response to PA on future PA behavior, one should expect that, consistent with psychological hedonism, a positive shift on the affective valence dimensions (greater pleasure or less displeasure) or either an increase in positive activation or a decrease in negative activation in response to PA behavior will lead to greater likelihood of future PA behavior. This idea – that people pursue pleasure and avoid displeasure – is a basic and ancient principle of human behavior and is referred to in the academic literature as “psychological hedonism” or the “hedonic principle” (Cabanac, 1992; Kahneman et al., 1997; Rhodes and Kates, 2015; Williams, 2018, 2019).

Another important factor when considering affective response to PA is the distinction between during-behavior and post-behavior affective response. As noted in the AHBF, this distinction is important because for many health behaviors the way one feels during behavior is markedly different and often has an opposite affective valence to how one feels immediately following the behavior (e.g., smoking or drinking lapses, drug use, and illicit sexual behavior). Indeed, while there is great variation in how individuals respond affectively during PA, affect post-PA is known to improve almost universally, this is known as the “affective rebound effect” (Ekkekakis, 2003; Ekkekakis et al., 2005, 2011; Jung et al., 2014; Cavarretta et al., 2019; Box et al., 2020).

### PA Intensity and Affective Response

While the focus herein is on PA as the dependent variable, factors that consistently determine affective response to PA are relevant to the extent that affective response to PA, in turn, determines PA behavior. The intensity of the PA stimulus is perhaps the factor most robustly associated with affective response to PA. Laboratory studies have demonstrated near-universal negative shifts in affective valence as PA intensity increases beyond the ventilatory threshold (VT); however, at intensities close to the VT, there is considerable intra-individual variation influenced by physical fitness, PA levels, body composition, and other cognitive factors (Ekkekakis et al., 2004, 2008).

According to Dual Mode Theory (Ekkekakis, 2003), shifts in affective valence in response to PA are determined by two orthogonal factors: cognitive parameters (e.g., self-efficacy, attitudes, and body image) and interoceptive cues (e.g., pain, temperature, and cortical oxygenation). Cognitive parameters influence affective valence at intensities below VT, such that, for example, holding more or less positively valenced affective attitudes about PA, or possessing more self-efficacy with respect to ability to perform PA may up- or downregulate affective valence, respectively. At intensities above VT and beyond, however, interoceptive cues dominate, evoking highly salient sensations of displeasure and overwhelming cognitive factors that may otherwise preserve or positively influence valence at lower intensities. Because the variability in affective response to PA most often occurs below VT, researchers studying affective response to PA typically set the exercise stimulus intensity to be at or near to a participant’s individual VT.

Briefly, it noteworthy that a number of studies have explored what effect completing high-intensity PA in discrete intervals across a workout [e.g., high-intensity interval training (HIIT)], rather than at a continuous pace, has on affective response to PA. Consistently, these studies find that affective response is more negatively valenced in response to the high-intensity conditions (both when the high-intensity is performed in intervals and steady state) than in the moderate-intensity conditions (Jung et al., 2014; Niven et al., 2018; Box et al., 2020). One study examined the affective-rebound effect mentioned above but applied examination of this phenomenon to the rest periods during interval PA training. Specifically, Box et al. (2020) found participants completing a HIIT session experienced greater increases in positively valenced affect between intervals compared to participants completing a moderate-intensity interval training session, but this spike was not large enough to account for declines (“plummets”) in affective valence during the interval sessions. Some research suggests that high-intensity PA might be more palatable if the intensity of the PA stimulus decreases over the course of the bout. For example, a study by Zenko et al. (2016) found that a “ramping down” approach (starting out with high intensity and declining toward the end of the bout) produced a positive affective response slope and yielded more favorable outcomes on post-PA affect processing constructs (i.e., remembered affect and anticipated affect) compared to a condition where intensity was increased over the course of the bout.

### Affective Response as a Putative Determinant of PA Behavior

A systematic review conducted by Rhodes and Kates (2015) summarized the extant literature regarding the relationship between affective response to PA and future PA behavior. They identified four studies examining during-PA affective response as a predictor of future PA (Williams et al., 2008, 2012; Schneider et al., 2009; Kwan and Bryan, 2010). Despite considerable variation in participant demographics (e.g., age and activity level), PA stimulus (e.g., cycle ergometer and treadmill), affect assessment [e.g., Physical Activity Affect Scale (PAAS; Lox et al., 2000), Feeling Scale (FS; Hardy, and Rejeski, 1989)], and PA assessment method (e.g., actigraphy and self-report), the relationship between affect experienced during PA and subsequent PA was positive in all four studies (Rhodes and Kates, 2015).

In two additional studies conducted after the Rhodes and Kates (2015) review, further support was shown for affective response to PA as a determinant of future PA behavior among low active adults but assessed outside of the laboratory *via* EMA. Williams et al. (2016) instructed participants (*N* = 59) to use a handheld device to indicate each time they were beginning a bout of PA over a 6-month period. Participants were then sent EMA prompts every 10-min while they completed the PA and then again 15-min post-PA termination. Small to medium effect sizes were observed for the association between affective response and the duration and latency of the next PA session. Liao et al. (2017a) used EMA to measure participants’ (*N* = 82) affect in response to PA when concurrent PA was also reported during a 4-day baseline period. These affect scores were then used to predict PA behavior, measured using accelerometry, 6- and 12-months later. The authors determined a 1-unit increase in positively valenced affect reported during concurrent PA was associated with an additional 4.62 min of PA per day 6-months later and an additional 5.24 min of PA per day 12-months later. Additionally, a 1-unit increase in negatively valenced affect reported during concurrent PA was associated with a decrease of 18.11 min of PA per day at 12-months, but the association between negatively valenced affect and PA at 6-months follow-up was not statistically significant.

In the Rhodes and Kates (2015) systematic review, nine studies were identified that examined post-PA affective response and future PA behavior (Berger and Owen, 1992; Klonoff et al., 1994; Annesi, 2002a,b, 2005; Schneider et al., 2009; Kwan and Bryan, 2010; Williams et al., 2012; Hargreaves and Stych, 2013). Again, there was significant variability among studies regarding covariates used (baseline affect and past behavior), sample characteristics (mixed PA experience background for adolescents and adults, sedentary adults, and new members to a fitness center) time of affect measurement, and type of affect measured. Here, only two of the nine studies found an association between immediate-post-PA affect and future PA (Berger and Owen, 1992; Annesi, 2005). Consistent with this general lack of positive findings, there was no association between post-PA affective response and future PA behavior in the EMA study conducted by Williams et al. (2016); post-PA affective response was not measured in the study by Liao et al. (2017a) discussed above.

The positive findings for during-PA affective response are consistent with psychological hedonism in that more positive (or less negative) affective responses during PA led to greater likelihood of future PA behavior. Interestingly, the hedonic principle, in its various incarnations, does not specify whether it is how one feels during or upon termination of the target behavior that is more likely to influence future behavior. However, the less robust findings for post-PA affective response are consistent with principles of operant conditioning in that more distal consequences of behavior (post-PA affective response) are less predictive of future behavior than more proximal consequences of behavior (during-PA affective response; see Williams, 2008).

A recent investigation, also conducted after the Rhodes and Kates (2015) review, sought to determine whether or not affective response to PA is a mechanism that can be improved (shifted more positively in valence) as a function of PA training volume completed over time and/or improvements in cardiorespiratory fitness (i.e., VO2max; Stevens et al., 2020). This line of research is predicated on past findings (from mostly cross-sectional study designs) that affective response to PA is more favorable among individuals who are more physically active and more physically fit (Parfitt et al., 1996; Reed et al., 1998; Bryan et al., 2007; Hallgren et al., 2010; Rose and Parfitt, 2012; Magnan et al., 2013; Frazao et al., 2016). Physically inactive participants (*N* = 201) were randomly assigned to one of four exercise training conditions fully crossed on intensity (moderate and vigorous) and duration (short and long). Training occurred over 16-weeks and in-bout assessments of affective response (Feeling Scale; Hardy and Rejeski, 1989) and perceived exertion [measured using the Rating of Perceived Exertion (RPE) scale; Heath, 1998] were collected during weeks 1, 4, 8, and 16. Results showed that across conditions, affective response to exercise did not change, on average, over 16-weeks despite associated improvements in cardiorespiratory fitness. Conversely, RPE decreased slightly, on average, over time. Further, while baseline affective response scores were positively associated with exercise minutes at follow-up, consistent with Rhodes and Kates (2015), average affective response scores collected across the intervention were not associated with minutes of exercise at follow-up.

### Future Directions

Research in this area consistently shows a positive association between more positively valenced affective responses during PA and future PA behavior. From a translational perspective, the next steps in this line of research are to develop intervention strategies that can result in positive shifts in affective valence in response to PA. As discussed, reducing the intensity of PA is a reliable strategy for improving affective response (Ekkekakis et al., 2011). Building off of this evidence base, and further informed by findings that preference and autonomy in the selection of PA intensity positively impact affective response (Lind et al., 2008; Rose and Parfitt, 2008; Vazou-Ekkekakis and Ekkekakis, 2009; Williams and Raynor, 2013), a growing number of studies have tested “self-selected intensity” or “affect-regulated” approaches to PA promotion (Parfitt et al., 2006; Costa et al., 2015; Williams et al., 2015, 2016; Baldwin et al., 2016).

While traditional PA prescriptions instruct participants to regulate PA based on certain thresholds for intensity (e.g., % of VO_2_max or % heart rate reserve; Garber et al., 2011), self-selected intensity PA prescriptions allow participants to choose their own intensity, whereas affect-regulated prescriptions explicitly instruct participants to choose an intensity that feels good (Zenko et al., 2017). Both self-selected intensity and affect-regulated prescriptions appear to yield more positive affective responses to PA (Parfitt et al., 2006; Lind et al., 2008; Williams et al., 2016) and greater PA engagement over time compared to traditional intensity-based PA prescriptions (Williams et al., 2015, 2016; Baldwin et al., 2016). Further, there is evidence that age and cardiorespiratory fitness moderate the effect of this approach on PA engagement such that it is most effective for individuals who are older and/or with lower cardiorespiratory fitness (Baldwin et al., 2016; Lee et al., 2020).

Another trend in this area of research is to conceptualize affective response to PA as a psychological or intermediate phenotype that mediates the effects of favorable PA genotypes on PA behavior (Bryan et al., 2017; Lee et al., 2018). This approach suggests that some individuals may be genetically predisposed to a more negative affective response to moderate-to-high intensity PA, whereas others may be predisposed to a more positive affective response. Rather than a more positively valenced affective response to PA resulting from higher activity levels/greater physical fitness – as the study by Stevens et al. (2020) explored and did not find evidence to support – certain individuals may be more drawn to PA (and thus become more physically fit) because they are genetically predisposed to a more positively valenced affective response to PA. Some individuals may possess this phenotype, but its expression could be masked or restricted due to the presence of other physiological factors known to influence affective response to PA such as BMI or aerobic deconditioning. Alternatively, expression might not be realized due to social-environmental factors that limit opportunities for PA engagement. Although work exploring genetic underpinnings are limited, at least two studies have identified genetic variants associated with affective response to PA (Bryan et al., 2007; Karoly et al., 2012).

Conceptualizing the affective response to PA as an intermediate phenotype could have implications for designing more targeted PA interventions. For example, for those individuals who are predisposed to a negative affective response to PA, interventions that emphasize distraction (Lind et al., 2009; Blanchfield et al., 2014; Gillman and Bryan, 2016), habit formation (Kaushal et al., 2017; Rhodes and Gray, 2018; Rhodes and Rebar, 2018), and/or contextual and situational modifications (Dunton et al., 2015; Stevens et al., 2016; Zenko et al., 2016) may be more effective; conversely, for those who are predisposed to a more positive affective response to PA, a mindful approach to PA (Cox et al., 2017; Edwards and Loprinzi, 2019; Gillman and Bryan, 2020) and/or pre-PA expectation manipulations (Helfer et al., 2015; Kwan et al., 2017) may be more effective.

It is not yet known what effect regular practice of strategies like distraction, situational modification, or mindfulness might have over time on the affective response to PA. At present, the effects of affective-response-improving strategies have only been tested in single-session or limited session formats. Thus, it is not known if these strategies would continue to bolster affective response if used consistently over time or whether habituation effects may be observed. Also unknown is whether *combined use* of strategies could produce markedly greater improvement in affective response over use of individual strategies or whether affective response would return to a participant’s baseline if strategies are not continued over time.

## Incidental Affect

Incidental affect refers to how one feels throughout the day. This type of affect is termed “incidental” because it occurs outside the context of the target behavior. Thus, incidental affect is not, like affective response, a direct result of the target behavior; however, incidental affect may influence engagement with the target behavior and/or be influenced by the target behavior (Williams and Evans, 2014). As with affective response, incidental affect constitutes affect *per se*; therefore, the same measures that can be used to measure affective response, for example, the Feeling Scale (Hardy and Rejeski, 1989), can be used to measure incidental affect. Incidental affect could be measured across the broad spectrum of affect as core affect, or as a specific affectively charged state, such as fatigue or anxiety, or as moods or emotions.

The majority of research evaluating the role of incidental affect as a determinant of health behaviors (beyond just the PA literature) has focused on how negative incidental affect influences behaviors that are harmful to health such as smoking, substance use, and binge eating (Williams and Evans, 2014). Two main theoretical perspectives – “affect regulation” and “affect congruency” theories – have informed much of the work in this area. As the name implies, “affect congruency” theories predict incidental affect will promote engagement with target behaviors that foster more similarly valenced affect, whereas “affect regulation” theories predict individuals will engage in behaviors that are expected to modify the current affective state.

With respect to negative incidental affect, affect regulation theories share conceptual overlap with principles of negative reinforcement (Williams and Evans, 2014) such that behavior is strengthened when associated with the removal or avoidance of an aversive stimulus (e.g., negative affect; Baker et al., 2004; Koob, 2013). This helps to explain why maladaptive behaviors (e.g., smoking, substance use, binge eating, and excessive sedentary time) are maintained even when they are incongruous with long term goal pursuits (e.g., smoking cessation, sobriety, weight loss, etc.) and/or no longer produce the same reward value (Tice et al., 2001; Volkow et al., 2010). In the context of PA, theories of affect congruence would predict that positive incidental affect is more likely to lead to PA behavior than negative incidental affect, whereas theories of affect regulation would predict that negative incidental affect may be more likely to lead to PA behavior if one expects that engaging in PA will produce a positive affective response.

### Negatively Valenced Incidental Affect

In terms of negatively valenced incidental affect preceding PA, both clinical and non-clinical populations report engaging in PA as an affect improvement strategy (Aldao and Dixon-Gordon, 2014; Jekauc and Brand, 2017) consistent with theories of affect regulation. Although, notably, among individuals with disordered eating pathology and/or high body image dissatisfaction, negative incidental affect often predicts PA engagement that is compulsory and performed despite illness or injuries (Goodwin et al., 2012b; Brownstone et al., 2013; Castonguay et al., 2017); thus, in such instances, PA may be functioning as an outlet for escaping negatively valenced affect more so than as an outlet for promoting positively valenced affect.

Outside the context of clinical populations, work in this area shows that incidental negatively valenced affect is associated with reduced likelihood PA will take place, consistent with affect congruency theories. A study conducted by Burg et al. (2017) used EMA methodology to measure affect (stress) experienced across the day as well as PA engagement over a 12-month period and found more stress experienced in the morning and/or previous evening resulted in 20–22% decreased odds of PA the subsequent day. Another recent EMA study conducted by Kerrigan et al. (2020) with participants enrolled in a behavioral weight loss trial found lower levels of negatively valenced affect and lower variability in negative affect, compared to one’s baseline, predicted greater PA the following day. In contrast to these findings, a prior systematic review examining acute (within a few hours) relationships between PA and affect (experienced in non-laboratory settings) found no significant associations between negatively valenced incidental affect and subsequent PA (among the six studies meeting criteria for review at the time; Liao et al., 2015).

### Positively Valenced Incidental Affect

Positively valenced incidental affect is theorized to increase approach motivation and influence engagement with a number of health behaviors, including PA (Fredrickson, 2004; Lyubomirsky et al., 2005; Catellier and Yang, 2013; Kim et al., 2017; Rhodes et al., in press). In the majority of work regarding the relationship between positively valenced incidental affect and PA, positive associations have been observed across studies with heterogeneous participant samples and a wide variety of study designs/methodologies (Audrain et al., 2001; Carels et al., 2007; Schondube et al., 2016). The English Longitudinal Study on Aging, a study involving nearly 10,000 older adults found “psychological wellbeing” (a broad conceptualization of positively valenced affect) to be independently associated with greater PA participation over an 11-year period (Kim et al., 2017). Interestingly, higher baseline psychological wellbeing was associated with a slower rate of PA decline over the 11-year period among participants who were already physically active at baseline; among participants who were inactive at baseline, PA engagement increased over time.

An experimental study conducted by Cameron et al. (2018) demonstrated that positively valenced incidental affect can positively influence PA goal striving and attainment. Specifically, participants were randomly assigned to a positive affective valence (joy) induction condition or a neutral affective valence condition and then given the choice of participating in 20-min of PA (walking) or 20-min of a sedentary activity. The authors found more participants assigned to the positive affective valence condition (55.17%) than the neutral affective valence condition (36.67%) elected to participate in the 20-min walking task and performed more PA during the task.

In another recent study, Emerson et al. (2017) recruited a sample of (*N* = 59) inactive, overweight/obese participants participating in a 6-month long PA intervention program and used EMA to collect multiple, random assessments of incidental affect and PA across the day for 29 consecutive days, and then again over 8-day segments 3- and 6-months into the PA intervention. Results demonstrated significant within-subjects cross-lagged effects: More positively valenced incidental affect at the start of a given day was associated with increased likelihood of PA later in the day (79% increased odds of PA associated with a 1 unit increase in affect assessed using the Feeling Scale; Hardy and Rejeski, 1989); and, after controlling for earlier incidental affect, PA participation was associated with more positively valenced post-PA incidental affect. The authors conclude that there is evidence of a reciprocal relationship between positively valenced affect and PA, but the strength of effect appears larger for the “affect-PA” pathway than the “PA-affect” pathway.

Using a similar methodological approach and analysis strategy, Liao et al. (2017b) found that results largely consistent with this those observed by Emerson et al. (2017). Specifically, the authors used EMA to measure participants’ (*N* = 110) incidental affect at eight random times throughout the day during a 4-day period and accelerometry to concurrently measure PA behavior. More positively valenced affect and less negatively valenced affect were positively associated with PA minutes completed over the subsequent 15 min and more PA was associated with more positively valenced affect over the subsequent 15 and 30 min.

### Future Directions

The findings summarized in this section suggest efforts to improve PA engagement may benefit from a focus on fostering more positively valenced incidental affect (and lower levels of negatively valenced affect) as well as potentially generating strategies for provoking “bumps” in positively valenced affect (especially at times across the day where affect may be vulnerable to negative shifts in valence). The proliferation of smartphone technology and methods, such as machine learning, hold significant promise regarding future opportunities for precision medicine in this area (Michie et al., 2017; Jacobson et al., 2019; Wilhelm et al., 2020). For instance, one could envision the development of a just-in-time intervention combining use of EMA and machine learning to anticipate participant behavior based on the micro-temporal associations between affect and PA (Nahum-Shani et al., 2016; Dunton et al., 2019).

Future work in this area will also need to address concerns regarding how to delineate post-PA affective response from post-PA incidental affect. Namely, how quickly does the acute affective response to PA dissipate and when is affect measured post-PA no longer considered influenced by the target behavior (and thus no longer considered affective response)? Are there individual differences regarding these phenomena? Do social, psychological, biological/physiological, or environmental characteristics moderate the speed of affective response dissipation or onset of incidental affective states? Can advances in sensor technology, EMA, and AI help to operationalize, capture, and differentiate between these affective states?

## Affect Processing

Whereas the first two categories of affect-related constructs (i.e., affective response and incidental affect) represent affect proper, affect processing constructs reflect cognitive processing of previous affective responses (Williams and Evans, 2014). The category of affect processing includes affective associations, implicit attitudes, affective attitudes, affective judgments, anticipated affective response (AAR), and remembered affect. Affect processing factors are distinct from affective response as they can be prompted any time outside the context of the target behavior, whereas affective response to a behavior (and incidental affect) can only be experienced “*in vivo*.”

Consistent with dual-process models, which posit that information about a stimulus or target is processed at two levels: one a quick, automatic, and intuitive level; the other a slower, deliberate, and reflective level (Smith and DeCoster, 2000; Evans, 2008; Kahneman, 2011; Reyna, 2012; Conroy and Berry, 2017; Brand and Ekkekakis, 2018) and affect processing constructs include both automatic (i.e., affective associations and implicit associations) and controlled processes (i.e., affective attitudes, anticipated affective response and remembered affect). Theoretically, in the reflective process, one first remembers their affective response, then anticipates a future affective response, and then forms an affective attitude. In the automatic process, memory and anticipation are automated and not separate steps – instead there are just affective associations that, in aggregate, lead to implicit attitudes. However, it is important to note that there is likely overlap between the two processing levels and corresponding affect processing constructs. For example, there is evidence that assessment of affective attitudes partly reflect implicit affective associations (Conner et al., 2011a).

### Affective Associations

Affective associations are defined as associations that exist in memory between PA and previously experienced affective responses to PA (Kiviniemi et al., 2007; Kiviniemi and Klasko-Foster, 2018). Although theoretically distinct from reflective affect processing constructs (Conroy and Berry, 2017), operationally, affective associations are assessed with self-report measures (e.g., “When I consider physical activity, I feel…”) that are quite similar to measures of affective attitudes (a reflective processing construct), in particular. As a result, the two factors may be difficult to distinguish empirically (Sala et al., 2016).

Research on affective associations as a determinant of PA is more limited than other affect processing factors. There is evidence that affective associations are associated with PA above and beyond known social cognitive predictors. In one study, Kiviniemi et al. (2007) asked healthy adults (*N* = 433) to report on various social cognitive beliefs relevant to PA, including their affective associations. Specifically, participants reported how they feel when considering PA (e.g., sorrow-joy) on three items. Participants also reported the number of vigorous PA minutes they engaged in per week. The results indicated that affective associations were significantly associated with vigorous PA minutes, after controlling for perceived benefits of PA, perceived barriers, social norms, and perceived behavioral control. In addition, a small pilot study (*N* = 19) tested the feasibility of targeting affective associations of PA *via* an evaluative conditioning approach delivered *via* smartphones (Conroy and Kim, 2020). Specially, positively-valenced PA images were installed as background on the phone lock screens to provide multiple daily exposures of the images to participants. This intervention approach resulted in increases in PA over 8 weeks (in a single group pre-post design), along with changes in several measures of PA-related affect, signaling this evaluative conditioning approach may be a promising way to target affective associations.

### Implicit Attitudes

Implicit attitudes are also defined as automatically triggered associations between two stimuli and are thus conceptually similar to affective associations. However, implicit attitudes are broader than affective associations in that implicit attitudes may or may not involve affect. Additionally, unlike affective associations, which are typically assessed *via* self-report, implicit attitudes are assessed *via* reaction-time tasks in which participants respond to PA-related word or image cues paired with affective descriptors (e.g., good-bad; see Conroy et al., 2010).

In a study of implicit attitudes and PA (Conroy et al., 2010), healthy young adults (*N* = 201) completed a reaction time-base implicit association test (IAT) to measure their implicit attitudes toward PA, along with other beliefs relevant to PA. They were given pedometers and their step counts were assessed over 1 week. Scores on the IAT predicted pedometer step counts above and beyond barrier self-efficacy and behavioral intentions. However, explicit measures of affective associations, or affective attitudes, were not included.

Taken together, evidence for the relationship between affective associations, or implicit attitudes, and PA is promising, but there is a need for additional evidence. Furthermore, there is need for future research to clarify whether implicit measures are capturing unique variance that is not captured in explicit measures of affective associations.

### Affective Judgments

Much of the research to date on affect processing factors involves affective attitudes and enjoyment. Affective attitudes are defined as evaluations of PA based on an aggregation of the likelihood and evaluation of affective outcomes of PA (e.g., the likelihood that PA will be enjoyable and how important that is to the respondent). This is in contrast to instrumental attitudes that are based on the same estimation of likelihood-evaluation but for instrumental outcomes (e.g., the likelihood that PA will reduce the risk of heart disease and how important that is to the respondent). While affective attitudes are theorized to be a function of separate likelihood and evaluation components, in direct assessment of affective attitudes respondents report the extent to which they evaluate PA using different affective descriptors (e.g., PA is enjoyable, boring, and pleasant). In the PA literature, the construct of PA enjoyment is most often operationalized and assessed with the Physical Activity Enjoyment Scale (PACES; Kendzierski and DeCarlo, 1991), a measure that is very similar in content to measures of affective attitudes in that it also prompts respondents to evaluate PA using different affective descriptors. Moreover, enjoyment has been examined jointly with affective attitudes in meta-analyses (Rhodes et al., 2009; Nasuti and Rhodes, 2013), under a broad umbrella term of affective judgments (Rhodes et al., 2019). For these reasons, we consider PA enjoyment in the same category as affective attitudes.

In the past 10 years, two meta-analyses conducted by Rhodes et al. (2009) have examined the association between “affective judgments” (i.e., affective attitudes and enjoyment) and PA behavior in adult samples and youth samples (ages 5–18; Nasuti and Rhodes, 2013). The meta-analysis on adult samples included 102 studies (114 independent samples) and the summary effect size was *r* = 0.42, a moderate sized effect and larger than effects reported in meta-analyses of the built environment (Duncan et al., 2005), socio-demographic (Bellows-Riecken and Rhodes, 2008), and personality (Bogg and Roberts, 2004; Rhodes and Smith, 2006) variables. The meta-analysis on youth samples (Nasuti and Rhodes, 2013) included 55 studies (70 independent samples), of which there were 15 experimental/intervention studies. The summary effect size for the correlational studies was *r* = 0.26 and for the experimental/intervention studies the effect size was *d* = 0.25, small-to-moderate effects. These effect sizes are smaller than in the meta-analysis with adult samples, perhaps due to PA behavior being more of a volitional behavior in adulthood compared to childhood and adolescence. In sum, the two meta-analyses on affective attitudes provide robust evidence that these factors are important correlates and determinants of PA behavior.

Interventions that target affective attitudes can be effective in influencing PA. Conner et al. (2011b) conducted two studies with young adult samples (*N* = 383 and *N* = 197) in which participants were randomized to read messages focused on (a) the affective benefits of exercise (e.g., it can reduce anxiety, depression, and stress) or (b) the instrumental benefits of exercise (e.g., it can prevent cancer and build healthy muscles and joints). There was also a no-message control condition. In both studies, the affective benefits message resulted in more exercise sessions over a 3-week period than the instrumental benefits message or no message control. Results from both studies also indicated that the intervention effect was partially explained by changes in affective attitudes. Additional findings suggested that the message intervention was more effective for individuals who tend to rely on emotion in decision-making (i.e., need for affect) and less effective for those who have a high need to think and deliberate on problems (i.e., need for cognition). Overall, the findings from these two experimental studies provide compelling evidence that intervening to focus people’s thinking and attention on affective aspects of exercise can more effectively influence exercise behavior than focusing on the instrumental aspects of exercise.

### Anticipated Affective Response

Anticipated affective response (AAR) is defined as the expectation of how one will feel in response to engaging in, or failing to engage in, PA. Conceptually, it is a type of outcome expectation, a construct found in most expectancy-value theories of health behavior (e.g., Health Belief Model, Social Cognitive Theory, and Theory of Planned Behavior). In the PA literature, short-term AAR has typically been assessed immediately prior to an exercise bout, with longer-term AAR assessed as anticipated affect in response to hypothetical future PA.

Studies examining short-term AAR have consistently demonstrated that AAR is positively associated with actual affective response during or shortly after the exercise bout (Ruby et al., 2011; Loehr and Baldwin, 2014; Helfer et al., 2015; Kwan et al., 2017). Two studies demonstrated this effect experimentally by manipulating what participants were told the exercise session they were about to complete would feel like, e.g., “most people report this activity feels good” and “most people report this activity does not feel very good” (Helfer et al., 2015; Kwan et al., 2017). Another consistent finding across these studies is that short-term AARs tend to underestimate how positive or pleasant the exercise will actually be (i.e., affective forecasting bias; see Ruby et al., 2011; Loehr and Baldwin, 2014), and this bias may be even larger among physically inactive individuals (Loehr and Baldwin, 2014). Despite the consistent effects AARs have on affective response during an exercise bout, it is important to note that the manipulation of anticipated affect did not have an effect on future PA behavior in the two experimental studies (Helfer et al., 2015; Kwan et al., 2017).

There is, however, stronger support for the effects of longer-term AARs on future PA behavior. Dunton and Vaughan (2008) asked participants to report their positive AARs (delighted, happy, fulfilled, calm, relaxed, and at ease) if they successfully engaged in regular PA over the subsequent 90 days, and their negative AARs (sad, dissatisfied, distressed, nervous, tense, and anxious) if they failed to engage in regular PA. Participants also reported their PA behavior at baseline and at 90 days follow-up. For those who were physically inactive at baseline, positive AAR but not negative AAR predicted those who were likely to adopt regular PA compared to those who stayed inactive. For those who were already physically active at baseline, the same pattern emerged in predicting the likelihood of maintaining PA over the 90 days. In sum, anticipated positive AARs associated with success were stronger predictor of future PA than anticipated negative AARs associated with failure.

Studies on anticipated regret as an AAR to failure to engage in future regular PA (Abraham and Sheeran, 2003, 2004) have demonstrated that anticipated regret influences future PA. Anticipated regret also positively influences the formation of PA intentions and explains unique variance in intentions not explained by past behavior or other social cognitive variables found in the Theory of Planned Behavior (Ajzen, 1991).

### Remembered Affect

Remembered affect is defined as the recall of the affective response during previous PA. Conceptually, remembered affect is distinct from affective response in that it is the *recall* of how one felt during a PA bout, whereas affective response is how one feels *while* engaging in PA. Remembered affect is also distinct from other affective processing factors in that it reflects cognitive processing of a specific PA bout rather than an aggregate evaluation of previous affective responses. Although there are limited data to date, the theoretical rationale for the importance of remembered affect on PA behavior is compelling. Specifically, Kahneman et al. (1993) have argued that it is our memories of affective response, rather than affective response *per se*, that determines whether we will be motivated to perform the behavior in the future (i.e., “remembered utility”; see Redelmeier et al., 2003).

Reports of remembered affect may be different than reports of experienced affective response as memory is prone to biases and often does not align with what was actually experienced (Broderick et al., 2008; Giske et al., 2010). For example, memory can be biased by cognitive heuristics, such as the peak-end rule (Kahneman et al., 1993), which suggests that memory is weighted more heavily toward affective experiences (e.g., affective response during exercise) that reflect the most intense (i.e., peak) and the end of the total experience.

Indeed, there is some evidence that remembered affect may deviate from actual affective response. Zenko et al. (2016) randomized participants to complete an exercise bout with either increasing or decreasing intensity. Ending an exercise bout more pleasantly than it began had a strong and enduring effect on how positively the bout was remembered 15 min, 24 h, and 7 days later. However, the effect of remembered affect on future PA was not examined. In a study described earlier by (Kwan et al., 2017) in which participants’ AARs were manipulated prior to an exercise bout, participants also reported their remembered affect following the exercise bout (“How do you remember feeling while exercising today?”) and were instructed to exercise every day for the upcoming week. Remembered affect was the only significant predictor of exercising all 7 days. Anticipated and experienced affective response during the bout did not predict PA. In terms of influencing PA behavior, remembered affect is a promising factor given evidence that people tend to base their deliberate decision-making about future behavior more on how they remember/recall their affective experiences rather the actual affective experiences (Kahneman et al., 1993; Redelmeier et al., 2003).

### Future Directions

An important future direction regarding affect processing factors is to clarify the inter-relations among affect processing variables, affective response, and behavior. Doing so would have important implications both for advancing understanding of their interrelations and for developing and refining novel interventions that can target these factors. The relations between affect processing factors and affective response are certainly reciprocal. AAR influences affective response during PA (at least when PA is performed at intensities below the VT), and affective response during PA influences how the PA is remembered and evaluated. While affective response during PA is theoretically the primary source of variance for the various affect processing variables, processed affect may also be dependent on other factors (environmental and social) that are tangential to the PA affect response (Rhodes et al., 2019). Further, it is not clear whether the effect of affective response during exercise on subsequent PA is mediated by remembered affect or another affective processing factor (e.g., affective attitudes and affective associations). If so, it would raise the possibility that interventions designed to target both affective response and the subsequent affect processing factor would be most effective.

A second future research direction in this domain is to clarify the extent to which measures of the different affect processing factors are measuring the same or different constructs (i.e., their construct validity) and whether they can be independently manipulated. Recent data suggest that there is a high degree of empirical overlap among the prevalent measures of affect processing factors (Chmielewski et al., 2016; Sala et al., 2016) and some of the measures have significant construct validity limitations. Addressing the construct validity of affect processing measures can clarify theoretical and empirical distinctions among the constructs (e.g., affective attitudes and enjoyment) and will improve the precision and reliability with which the constructs are assessed, which will also impact the design of interventions intended to modify them.

## Affectively Charged Motivation

As defined by the AHBF, affectively charged motivation for PA includes motivational states that have their basis in past affective responses to PA, such as craving, desire, dread, intrinsic motivation, and fear (Williams and Evans, 2014). Affectively charged motivation constructs differ from reflective motivation constructs, such as intentions and goals, which are a function of more deliberate consideration of the potential outcomes of a behavior. Consistent with the emphasis herein on affective determinants of PA, this section provides an overview of affectively-charged motivation to the exclusion of reflective motivation.

In the PA literature, intrinsic motivation, which has largely been studied in the context of Self-Determination Theory (SDT; Ryan and Deci, 2007), has received the most attention from among the affectively charged motivational states. A construct closely related to fear, anxiety sensitivity, also fits within the broad category of affectively charged motivation and will also be reviewed in this section. Finally, craving, desire, and dread collectively represent “hedonic motivation,” which has not yet received broad empirical support as an affective determinant of PA, but is likely to be important for understanding PA behavior (Williams, 2018, 2019; Williams and Connell Bohlen, 2019).

### Intrinsic Motivation

As posited in SDT, intrinsic motivation is the propensity to seek out pleasure, novelty, aesthetics, and spontaneous interest – it is motivation to perform a behavior or action for the inherent enjoyment that doing so provides (Ryan and Deci, 2000a). Intrinsic motivation for PA is often assessed with the Intrinsic Motivation Inventory (Mcauley et al., 1989) which asks respondents to rate their agreement with items such as, “I enjoyed this activity very much,” “this activity was fun to do,” and “I thought this activity was quite enjoyable.” Likewise, the Behavioral Regulations in Exercise Questionnaire (Mullan et al., 1997) includes items, such as “I get pleasure and satisfaction from participating in exercise,” “I exercise because it is fun,” and “I find exercise a pleasurable activity.” In contrast, extrinsic motivation is the propensity to perform a behavior or action in order to obtain external rewards or desirable outcomes (Ryan and Deci, 2000a). For example, being motivated to perform the same actions because one expects the experience will result in weight loss or improved social status. In this case, such an outcome may cause a person to feel good, but the motivation to perform the behavior is driven by the expected results of the behavior, not the experience of the behavior itself. Thus, intrinsic motivation pertains to the pursuit of affective outcomes of behavior whereas extrinsic motivation pertains to the pursuit of instrumental outcomes of behavior (Williams and Evans, 2014). Notably, because measures of intrinsic motivation necessarily ask respondents to rate their enjoyment of PA, some have argued that these scales unintentionally assess a construct that is essentially PA enjoyment (Rhodes et al., 2019); however, in theory, intrinsic motivation is a separate construct from PA enjoyment.

According to SDT, intrinsic motivation is the most autonomous form of motivation and is a driver of behavioral persistence. Consistent with this prediction, the overwhelming majority of evidence suggests that intrinsic motivation plays an important role in PA behavior and especially maintenance of PA behavior over time (Hagger and Chatzisarantis, 2007; Ryan and Deci, 2007; Standage et al., 2008; Wilson et al., 2008; Teixeira et al., 2012). As might be expected, individuals who engage in PA more frequently report stronger intrinsic motivation for PA (Teixeira et al., 2012), but inactive individuals have the capacity to develop increased intrinsic motivation for PA over time (Rodgers et al., 2010). As described by the Multi-Process Action Control Framework (Rhodes, 2017), increased exposure to PA and increased intrinsic motivation for PA lead to a stronger sense of self as “an exerciser” and this exercise identity promotes PA maintenance as PA behaviors become integrated into one’s daily routine (Gillman et al., 2017).

Consistent with the conceptualization of intrinsic motivation as the pursuit of affective outcomes of behavior, a study by Schneider and Kwan (2013) found that affective response to acute bouts of PA positively predicted intrinsic motivation for PA among a sample of adolescents. In another study, Schneider (2018) showed that intrinsic motivation mediated the relationship between affective response to an acute bout of PA and PA levels at baseline (cross-sectionally) and 5-months follow-up. Related research has shown that focusing on the proximal outcomes of PA, such as improved feelings states, may generate more intrinsic motivation for PA than focusing on distal outcomes (which are inherently extrinsically oriented), such as improved health (Evans et al., 2014).

### Fear and Anxiety Sensitivity

Fear is an emotion that motivates movement away from behaviors that have previously been associated with immediate negatively valenced affect (displeasure). For example, if past experiences of PA have been unpleasant or aversive, fear of experiencing those affective states again would motivate one to avoid PA. However, in rare circumstances, fear motivates PA as a means to an end (e.g., running away from something dangerous). While fear has received little attention with respect to its relationship to PA, anxiety sensitivity, sometimes referred to as “fear of fear” (Craske and Barlow, 2014), has been more thoroughly investigated.

Anxiety sensitivity is the fear of somatic arousal-related sensations commonly experienced when one is experiencing anxiety or panic, for instance increased heart rate, labored respiration, muscle tension, and sweating, because of a misappraisal of these somatic sensations as dangerous (Reiss and McNally, 1985). Anxiety sensitivity is one factor that may contribute to poor tolerance of negative affect/displeasure during PA, and ultimately, avoidance of PA (Smits et al., 2010; Sabourin et al., 2011; Moshier et al., 2013, 2016; Hearon et al., 2014).

Predictably, there is an observed inverse relationship between anxiety sensitivity and PA engagement. For instance, Moshier et al. (2016) found that anxiety sensitivity, measured using the Anxiety Sensitivity Index, a 16-item self-report measure including items such as “it scares me when I feel faint,” prospectively predicted PA engagement (negatively) over and above the effect of past PA behavior among a sample of college students who self-identified as being motivated to increase their PA participation. Interestingly, other theory-informed self-regulatory constructs, such as goal setting, action planning, and perceived behavioral control, were not predictive of PA at follow-up controlling for past PA. Although this study was conducted with a sample of young and healthy college students, the authors argue that the findings underscore the importance of examining affective factors, in this case anxiety sensitivity, with respect to the PA intention-behavior gap (Sheeran and Webb, 2016). In a prior study, Moshier et al. (2013) found a negative cross-sectional association between anxiety sensitivity and participation in vigorous intensity PA but no association between anxiety sensitivity and moderate intensity PA.

Others have extended this research to understand the role of anxiety sensitivity among individuals higher on BMI who have been shown to have a more negative affective response to PA (Ekkekakis and Lind, 2006). In a study by Smits et al. (2010), participants with various BMIs and levels of anxiety sensitivity were randomly assigned to 20 min of treadmill running at 70% of their age-adjusted predicted maximum heart rate or 20 min of rest; for both conditions, perceived distress measures were collected every 4 min. The researchers observed a significant condition by BMI by anxiety sensitivity interaction suggesting that higher levels of distress were experienced among participants higher on BMI, but only if they were also high on anxiety sensitivity (Smits et al., 2010). Similarly, Hearon et al. (2014) found that high anxiety sensitivity was associated with less engagement in moderate intensity PA over the course of 1 week for high BMI participants, but the inverse was true for participants with a BMI in the normal weight range.

One mechanism through which anxiety sensitivity may influence PA is through overestimation of PA intensity. For example, one recent study found participants with panic disorder reported significantly higher perceived exertion (measured as RPE; Heath, 1998) in response to PA during an exercise stress test compared to a group of healthy controls (not meeting clinical criteria for panic disorder) matched on age, BMI, and activity level (Muotri et al., 2017). Among the control participants, there was a clear positive correlation between RPE scores and percent of max heart rate, as is commonly reported in the literature, but among participants with panic disorder there was no such relationship. Further, participants with panic disorder achieved lower peak oxygen consumption (VO_2_max) scores during the exercise stress test compared to controls despite there being no differences between groups on activity level prior to exercise testing (all participants were predominantly sedentary). The authors speculate that participants with panic disorder are unwilling to push themselves to the point of exhaustion during testing due to poor tolerance of their autonomic experience (i.e., anxiety sensitivity; Muotri et al., 2017).

### Hedonic Motivation

The recently introduced concept of hedonic motivation is posited as the mechanism through which past affective experiences influence future behavior, consistent with the ancient and intuitive principle of psychological hedonism (Williams, 2018, 2019). Through the process of associative learning, behaviors that elicit immediate affectively favorable responses (e.g., pleasure or relief from displeasure) tend to become targets of hedonically driven craving/desire, whereas behaviors that elicit affectively unfavorable responses (e.g., displeasure) tend to become targets of hedonically driven dread, with hedonic craving/desire and hedonic dread representing opposite poles of hedonic motivation. Importantly, though experienced consciously, hedonic motivation is produced automatically without deliberate cognitive or affect processing regarding how the target behavior is expected to feel (AAR), how it was experienced previously (remembered affective response), or how performing the behavior may yield future costs or benefits (Williams, 2018, 2019). Consistent with incentive salience theory (Berridge and Robinson, 2003; Berridge, 2007), hedonic motivation and affective response are propagated by separate neurobiological pathways and feature distinct psychological characteristics (Williams, 2018, 2019). For instance, while the experience of pleasure is positively valenced, the experience of craving/desire may or may not be associated with positive valance; rather, craving/desire may be experienced as negative in valence, especially if the target behavior is unavailable (e.g., no desserts to eat in the immediate environment) or inconsistent with one’s goals or values (e.g., weight loss; Williams, 2018, 2019).

In the context of PA research, hedonic dread is more relevant than hedonic craving/desire because the public health problem related to PA is that people do not engage in enough of the behavior, rather than engaging in too much of the behavior (e.g., eating, smoking, alcohol and drug use, and risky sexual behavior). Hedonic dread of PA refers to the automatic subjective experience of not wanting to engage in PA and is a function of prior experiences of displeasure or discomfort during PA (Williams and Connell Bohlen, 2019). For many individuals who may stand to benefit greatly from increased amounts of PA, specifically those who are more overweight, sedentary, at risk for chronic illness, or living with chronic illness, affective response to PA is negative in valence, especially at more vigorous levels of intensity (Ekkekakis and Lind, 2006; Ekkekakis et al., 2011). While there is now a substantial literature on affective response to PA, there is, as yet, no research on the hedonic dread that represents the proximal linkage between previous negative affective response to PA and future avoidance of PA behavior. The concept of hedonic dread, in combination with the burgeoning research based on negative affective response to PA, provides a compelling rationale for why PA is a behavior that, despite providing extensive of health benefits, is plagued by poor adherence and drop out (National Center for Health Statistics, 2018).

Hedonic craving/desire is, in the context of PA, most relevant for research on pathological dependence on PA. Both craving and desire refer to the automatic subjective experience of wanting to perform a behavior. “Exercise dependence” has been defined in terms of “craving” for exercise that is excessive and intractable despite compromised health, wellbeing, and/or social and occupational role functioning (Hausenblas and Downs, 2002; Chen, 2016; Macfarlane et al., 2016; Lichtenstein et al., 2017; Marques et al., 2019). Exercise dependence is not a common condition; approximately 0.5% of the general adult population are believed to meet criteria (Chen, 2016), but a study by Lichtenstein and Jensen (2016) found that the prevalence among CrossFit participants (a high intensity strength and conditioning fitness program) may be as high as 5%. Although exercise dependence and other similar terms (e.g., “exercise addiction,” “obligatory exercise,” and “excessive exercise”) are defined by the presence of intense “craving/desire” for exercise, little to no research has been conducted to directly measure craving/desire for PA. Rather, much of this literature focuses on the measurement of exercise dependence and the relationship of the construct to other diagnostic or personality characteristics (Hausenblas and Downs, 2002; Hausenblas and Fallon, 2002; Hausenblas and Giacobbi, 2004; De Young and Anderson, 2010; Paradis et al., 2013; Holland et al., 2014; Hill et al., 2015).

### Future Directions

According to the AHBF, affectively charged motivational states are posited to be the mechanism through which prior affective response to PA influence future PA. However, research testing this causal pathway is needed. Likewise, the AHBF suggests that affect processing constructs also influence hedonically charged motivational states, and thus, future work might explore whether the pathway from affective response to affectively charged motivation is mediated by affect processing constructs. Future work may also explore whether efforts to influence affectively charged motivational states are more effective if they do so by targeting affective response and affect processing factors simultaneously or separately.

Research on increasing intrinsic motivation for PA has begun to emphasize mindfulness techniques (Gardner and Moore, 2012). The notion that persons are motivated to pursue actions and activities, such as PA, that assist them in living a values-aligned life is a central tenant of acceptance-based approaches and is very near in concept to intrinsic motivation (Ryan and Deci, 2000b, 2007; Teixeira et al., 2012). According to practitioners of acceptance-based approaches, by leveraging the desire to live a values-aligned life and drawing out discrepancies between aspirations and actions (for instance, an individual who reports valuing her longevity, vitality, and/or ability to function independently into older age but is being unwilling to engage in PA due to associated unpleasant feelings), it may be possible to build self-identification with PA and increase intrinsic motivation for PA (Stevens and Bryan, 2015). Likewise, the concept of exposing oneself to different – potentially aversive – experiences and experiential contexts (e.g., the presence of uncomfortable feelings or sensations such as muscle cramping or shortness of breath during PA) is also an integral component of acceptance-based therapies and behavioral interventions. Acceptance-based approaches promote the development of mindful awareness and psychological acceptance skills that may increase willingness to experience and tolerate discomfort during PA (Stevens and Bryan, 2015), and indeed, evidence suggests that acceptance-based interventions show promise for increasing PA behavior (Ulmer et al., 2010; Butryn et al., 2011, 2018; Goodwin et al., 2012a; Kangasniemi et al., 2015; Pears and Sutton, 2020).

Finally, efforts to mitigate the effects of anxiety sensitivity on PA behavior may be informed by empirically supported treatments for anxiety and panic. For example, interoceptive exposure is a technique included in cognitive behavioral treatment approaches for anxiety that is used to help individuals stop avoiding behaviors or situations that elicit arousal symptoms (i.e., symptoms of a panic attack), such as climbing stairs, or jogging (Craske and Barlow, 2014). Interoceptive exposure works by repeatedly and purposefully eliciting arousal symptoms (i.e., sweating, elevated heart rate, and breathing rate) that are harmless, but feared, in a therapeutic context where escape/avoidance behavior can be mitigated. When treatment is successful, the individual learns through the exposure process that those symptoms are not actually dangerous and the fear response is extinguished. Some research has shown that PA can serve as the interoceptive exposure intervention to reduce anxiety sensitivity (Broman-Fulks et al., 2004; Broman-Fulks and Storey, 2008). Therefore, intervention work in the PA literature may be able to reduce avoidance of PA *via* “exposure” training sessions that help participants to experience somatic symptoms without terminating the activity.

## Summary and Conclusions

This conceptual review covered examples of affect-related correlates and determinants of PA in each of the four areas outlined by the AHBF: affective response to PA, incidental affect, affect processing, and affectively charged motivation. The majority of research concerning affect-related correlates and determinants of PA has been done in the areas of affective response and affect processing. The literature on incidental affect is smaller but growing. Less work has explicitly focused on affectively charged motivational states as correlates and determinants of PA, with the bulk of research in that area on the concept of intrinsic motivation.

Within each of the four AHBF categories, there is considerable heterogeneity in terminology, conceptualization, and assessment. This heterogeneity can lead to confusion and misinterpretation of research findings. For example, inconsistent use of the terms “positive affect” and “negative affect” as either labels for the dimensions of Watson and Tellegen’s (1985) the rotated circumplex model or as labels for positive vs. negative valence has led to considerable confusion in the literature (Ekkekakis, 2013). As research on affective determinants of PA evolves, researchers should strive to use terminology and conceptualizations for affect-related constructs that clearly delineate the constructs under study and distinguish them from related, but distinct constructs.

As a corollary to the latter point, more research is needed that examines the empirical distinction among different affect-related constructs, how they differentially predict and combine to predict PA behavior. In addition to prediction of PA, research is needed to elucidate the pathways through which different affect-related constructs inter-relate to influence PA behavior. Such research requires experimental designs and multiple assessments with appropriate temporal ordering of constructs to model potential causal pathways.

While the present paper has focused on affect-related factors, as highlighted in the AHBF, research is also needed that examines how affect-related (reviewed herein) and traditional cognitive factors (e.g., instrumental outcome expectancies and attitudes, social norms, self-efficacy, and behavioral intentions) interrelate to influence PA behavior. For example, when will affective factors matter most, and when will cognitive factors matter most? One possibility is that affective factors are central to determining motivation for PA, but specific plans and strategies are important for executing the behavior, with these affective and cognitive processes interacting on a daily basis. This idea is, however, merely conjecture at this point, as more research is needed.

Ultimately, what is needed is translation of the accumulating basic science findings on affect-related determinants of PA into targeted interventions to promote PA. For example, research is needed that identifies multiple strategies for influencing each type of affect category and then identifies which strategy or which combination of strategies is most effective at influencing the target affect category. Interventions could then be developed to target multiple levels of the AHBF and/or see how an intervention targeting one level of the ABHF “spreads” to other areas. For example, an evaluative conditioning intervention intended to impact affect processing may additionally show impact on affective response and/or affectively charged motivational states.

This review has highlighted – in the context of the AHBF – the many affect-related constructs that have been conceptualized and studied as putative determinants of PA behavior. As with cognitive constructs that have been studied over the past several decades, these affect-related constructs require in-depth research to understand the many ways in which they inter-relate to influence PA.

## Author Contributions

CJS and DMW conceptualized and drafted most of the manuscript, and finalized the manuscript. ASB, ADB, MC, and RER contributed subsections and provided feedback on the full manuscript. All authors contributed to the article and approved the submitted version.

### Conflict of Interest

The authors declare that the research was conducted in the absence of any commercial or financial relationships that could be construed as a potential conflict of interest.
